# Role of Strauss ECG criteria as predictor of response in patients undergoing cardiac resynchronization therapy

**DOI:** 10.1186/s43044-022-00308-3

**Published:** 2022-09-30

**Authors:** Khaled Ashraf Shoman, Hayam Mohammed Eldamanhory, Emad Effat Fakhry, Haitham Abdelfatah Badran

**Affiliations:** Cardiology Department, Ain Shams University, B6 – Madinaty-New Cairo, Abbassya, Cairo, 19519 Egypt

**Keywords:** Cardiac resynchronization therapy, Ejection fraction, Electrocardiogram, End-systolic volume, Global longitudinal strain, Global circumferential strain, Left bundle branch block, New York heart association

## Abstract

**Background:**

Cardiac resynchronization therapy (CRT) is a standard treatment in patients with heart failure; however, approximately 20–40% of recipients of (CRT) do not respond to it based on the current patients’ selection criteria. The purpose of this study was to identify the baseline parameters that predict the CRT response and how the ECG morphology can affect the outcome. The study aimed to evaluate the Strauss ECG criteria as a predictor of response in patients undergoing cardiac resynchronization therapy.

**Results:**

Out of 70 patients, 3 patients missed the 6-month follow-up after CRT implantation, so the study enrolled 67 patients that have been classified according to ECG morphology of LBBB to 37 patients with non-Strauss ECG criteria—one of whom died after 4 months—and 30 patients with Strauss ECG criteria. The number of responders in the study was 50 patients with percentage 75.8%; 52% of CRT responder (26 patients) had non-Strauss ECG criteria, while 48% of CRT responders (24 patients) had Strauss ECG criteria with *P* value = 0.463. While there was no statistical significance of overall CRT response nor 6-month hospitalization and mortality between patients of Strauss and non-Strauss ECG criteria, there was a significant improvement in NYHA class, EF assessed by biplane Simpson’s, end-systolic volume, global longitudinal strain and global circumferential strain by speckle tracking echocardiography in patients with Strauss ECG criteria of LBBB.

**Conclusions:**

There is no statistical significance in overall CRT response nor the 6-month hospitalization and mortality after 6 months of follow-up between patients with Strauss and non-Strauss ECG criteria of LBBB; however, patients with Strauss ECG criteria have better improvement in NYHA class, echocardiographic parameters such as EF and ESV and speckle tracking parameters (GLS and GCS).

## Background

Cardiac resynchronization therapy (CRT) is considered a cornerstone in treatment of chronic heart failure (HF) patients with left bundle branch block (LBBB) [1, 2]

European and American guidelines recommend CRT implantation as class one indication in symptomatic HF patients with QRS duration (QRSd) > 130 ms and LBBB morphology. However, the percentage of non-responders in patients with LBBB reaches up to 20–40% [3].

Different electrocardiographic (ECG) criteria for the diagnosis of LBBB may affect the response to CRT implantation [4].

The LBBB criteria for clinical practice in the American Heart Association (AHA) guidelines differ from those in the European Society of Cardiology (ESC) guidelines, but both of them recommend the absence of q waves in leads I, V5, and V6 [5].

In 2011, Strauss et al. proposed new stricter LBBB criteria to improve the CRT response, including a QRS duration of > 140 ms for men and > 130 ms for women, along with mid-QRS notching or slurring in at least 2 of the leads V1, V2, V5, V6, I, or aVL and also rS or QS morphology in lead V1 [6].

Actually, it remains an area of debate whether Strauss LBBB criteria could be more helpful than other criteria in predicting the CRT responders.

The baseline QRS duration is a cornerstone indication of cardiac resynchronization therapy. It has been established since the publication of Pacing Therapies in Congestive Heart Failure (PATH - CHF) I and II studies [7].

It was subsequently proved that the LBBB subgroup patients (approximately 70% of the total MADIT-CRT population) received great benefit from CRT-D implantation, while non-LBBB patients did not show the same results [3]. After MADIT-CRT study publication in June 2009, FDA requested extra 6 months of follow-up to assess the persistence of the beneficial effect of CRT-D over time.

In Sweeney et al. [8] study revealed that LBBB morphology is a strong predictor of response to CRT. This was further confirmed in the RAFT study by Lidija Poposka study [9].

LBBB configuration in the ECG was considered as the most significant predictor of the CRT response [10].

The presence of notching is very important to diagnose LBBB, and it should start just after 40 ms of the QRS, but before 50% of total QRS duration, when the activation wave-front reaches the endocardium of the LV [11].

In 2015, Jan Steffel revealed in Echo CRT trial results that patients underwent CRT implantation with QRS duration between 120 and 130 ms showed no benefit from implantation in the primary and secondary outcomes and that changed the ESC guideline for CRT implantation in heart failure patients to at least 130 ms not 120 ms [12].

Some of the studies pointed that patients with IVCD did not respond well to CRT implantation [12]. The study of Takaya et al. [13] revealed that only 40% of patients with IVCD responded to CRT. This response rate was low compared to large major trials, and the study revealed that patients with IVCD had fewer benefits from CRT therapy regarding symptomatic benefit and echocardiographic parameters [14, 15]

Inconsistent with the AHA criteria and the ESC criteria, Strauss et al. suggested that the absence of *q* waves in leads I, V5, and V6 is not included in the LBBB definition.

Besides, the QRS scoring system revealed that presence of q waves in those leads may be an indicator of presence of apical anterior myocardial infarction and cardiac fibrosis [16, 17]. But in 2012, Perrin et al. suggested that presence of q waves in those leads was better considered as a form of intraventricular conduction defect, thus it was associated with poor response to CRT [17].

The aim of this study is to evaluate the Strauss ECG criteria as a predictor of response in patients undergoing Cardiac resynchronization therapy.

## Methods

It was a prospective study with 6-month follow-up that included 70 patients with LBBB morphology who underwent CRT or CRT-D implantation following ESC guidelines of diagnosis and management of acute and chronic HF 2016 about CRT implantation (symptomatic patients with HF in sinus rhythm with LVEF ≤ 35, QRS duration ≥ 130 ms and LBBB morphology despite OMT) [18] in XXX, Cardiology department, from March 2020 to March 2022.

The approval of the xxx university ethical committee was obtained as it informs to the ethical guidelines of the 1975 declaration of Helsinki as revised in 2008.

Consent for participation and publication: All patients were informed about the study and a written informed consent was obtained for their participation and publication of their information.

The exclusion criteria: patients with heart failure symptoms but not fulfilling any criteria of CRT implantation, patient who underwent cardiac resynchronization therapy implantation with non-LBBB morphology, patient not achieving biventricular pacing 99% in the 6-month follow-up programming, patients who had periprocedural complications during CRT implantation such as pocket hematoma, pneumothorax, hemothorax, coronary sinus dissection and pericardial tamponade, patients with persistent AF, and patients with poor image quality pre- or post-CRT implantation with LBBB morphology were excluded from the study for the difficulty of doing endocardial tracing and speckle tracking analysis. Patient with previous right ventricle pacing were excluded from the study.

**Fig. 1:**
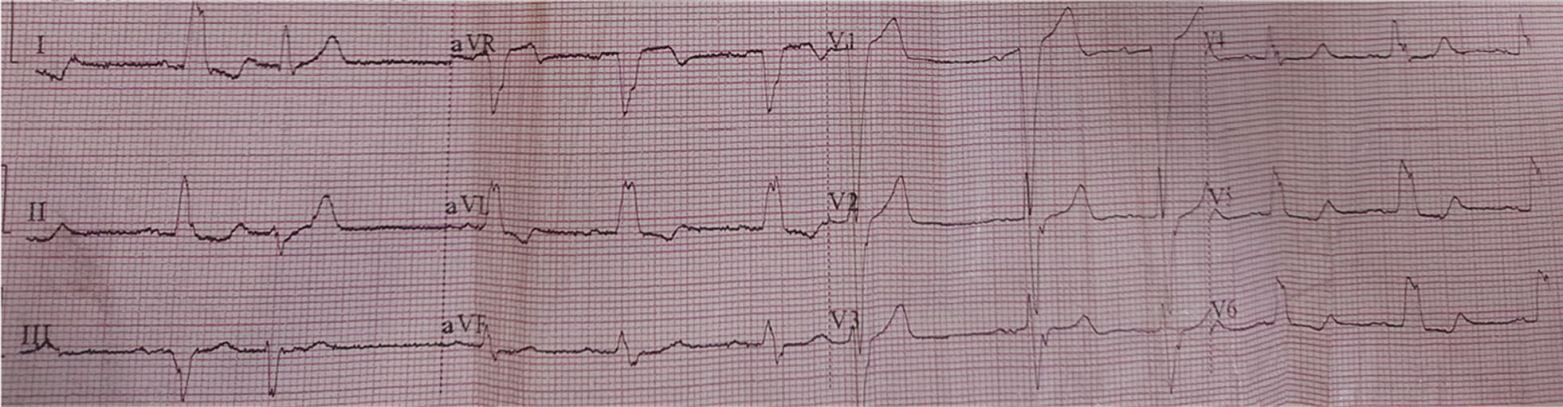
Example of Strauss ECG criteria of non-responder patient, notice notching in QRS complex in Lead I-aVL with QRS duration 140 ms

**Fig. 2:**
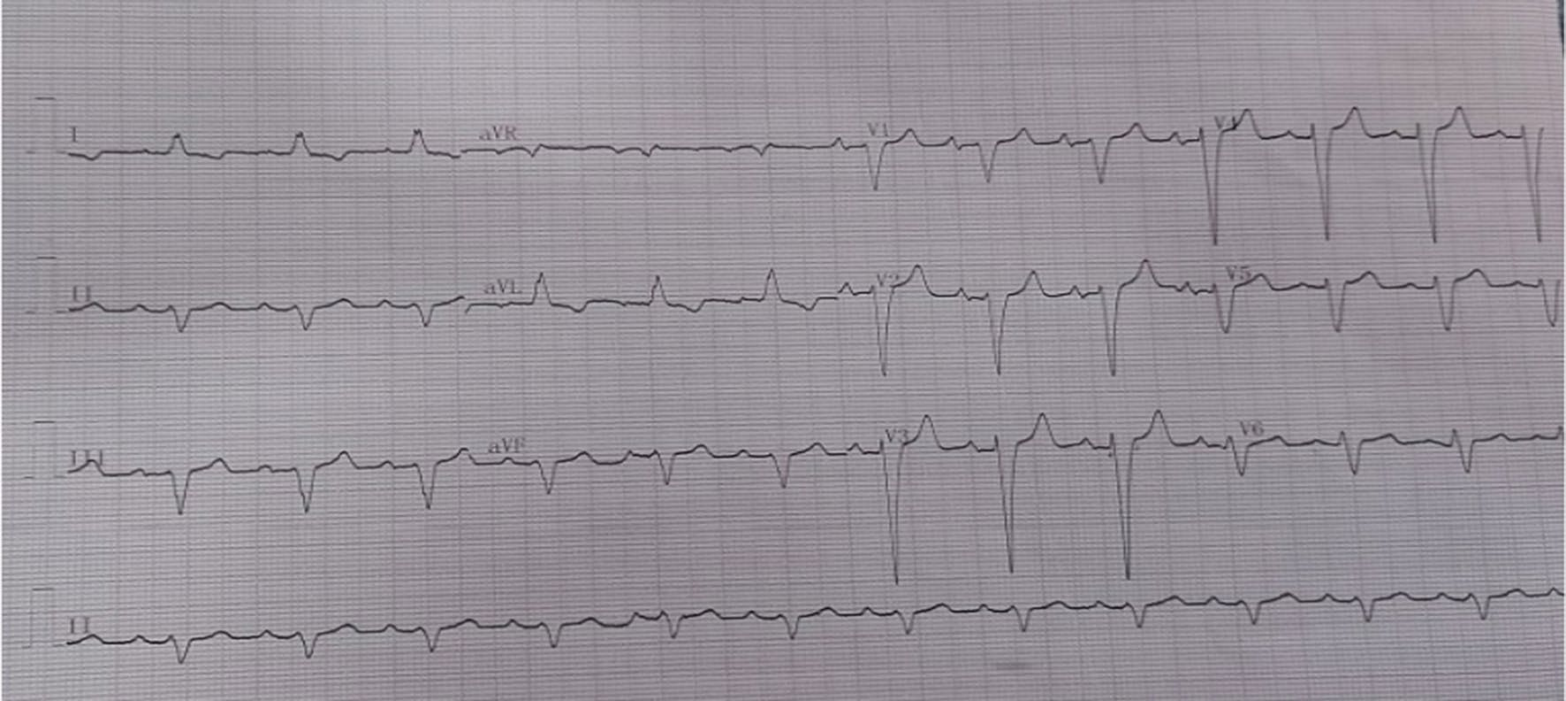
Example of Non- Strauss ECG criteria for responder male patient, QRS duration = 130 ms

The QRS morphology of the patients with LBBB-like pattern was analyzed, and patients were classified into two groups: Strauss LBBB criteria (SLBBB) group for patients with (a QRS duration of > 140 ms for men and > 130 ms for women, along with mid-QRS notching or slurring in at least 2 of the leads V1, V2, V5, V6, I, or aVL and also rS or QS morphology in lead V1 patients) **(**Fig. 1**)** and the non-Strauss LBBB (non-SLBBB) group for patients not fulfilling the previous criteria [2, 3] (Fig. 2).

All patient were subjected to proper history taking including (age, sex, coronary risk factors, ischemic etiology which was defined as stenosis ≥ 50% in left main coronary vessel or stenosis ≥ 70% in any other coronary vessel by MSCT coronary angiography or coronary angiography upon taking patient history or revising patient's documents, NYHA class of dyspnea before and after 6 months of CRT implantation and 6-month hospitalization and all-cause mortality as primary endpoint, clinical examination and 2D echocardiography and speckle tracking were done before and after 6 months of CRT implantation.

*CRT implantation* RV leads were placed at the RV apex, atrial leads were placed in the right atrial appendage, and LV leads were inserted in the lateral or posterolateral vein.

**Fig. 3:**
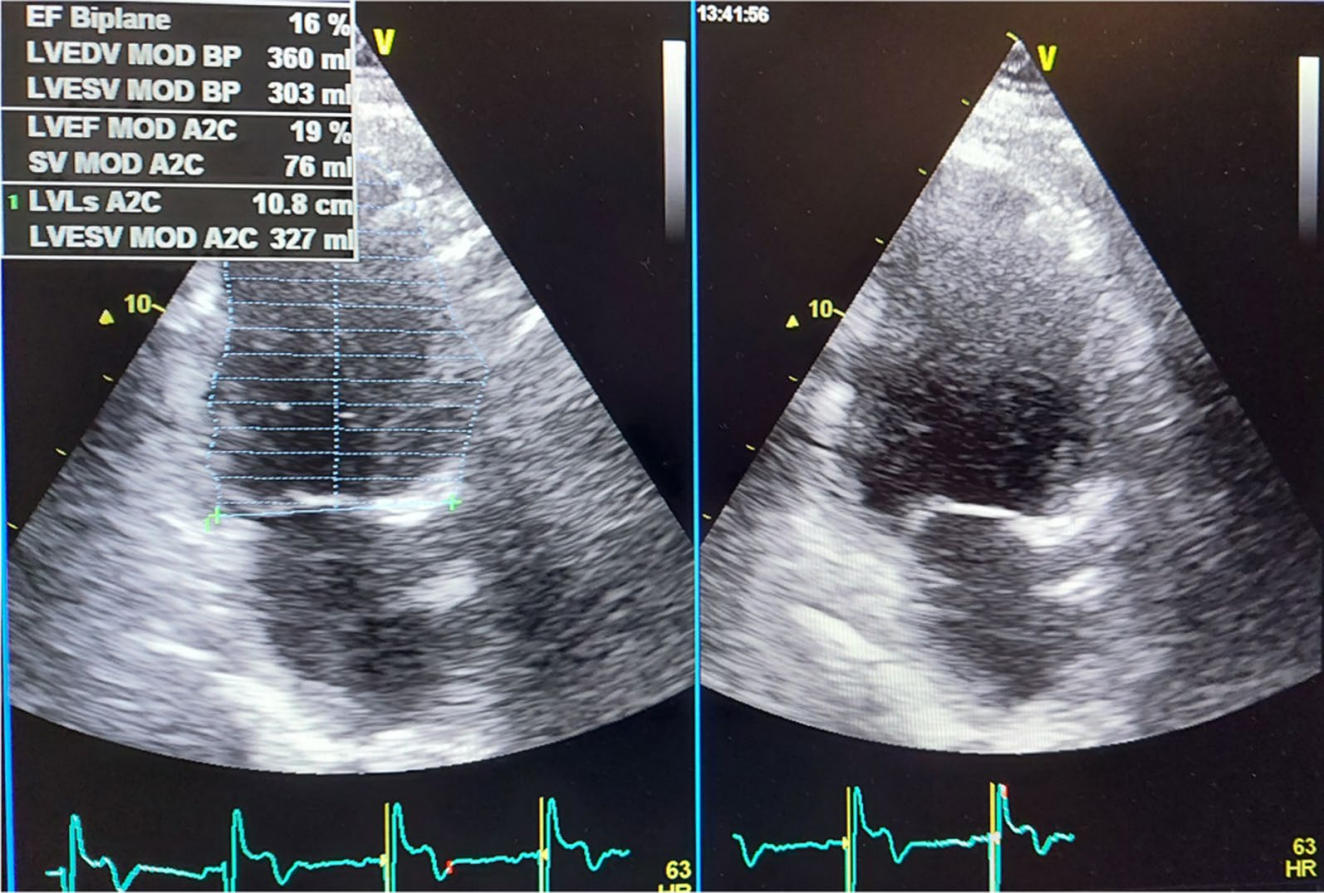
EF assess in the same patient of figure one before CRT implantation

**Fig. 4:**
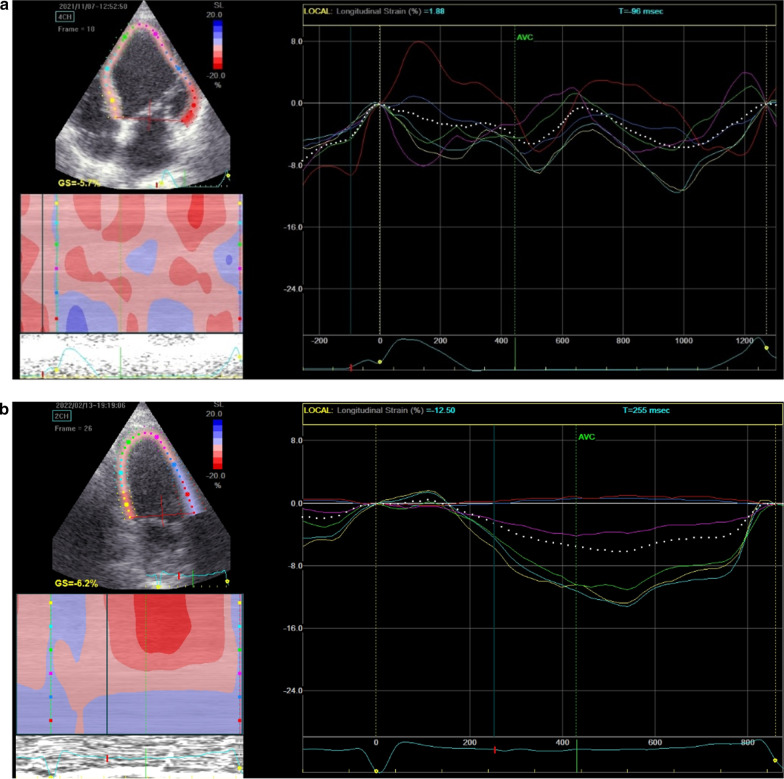
**a** Average GLS assessment for Strauss patient before CRT implantation. **b** Average GLS assessment for the same Strauss patient in Fig. 4 after CRT Implantation with no significant improvement

**Fig. 5:**
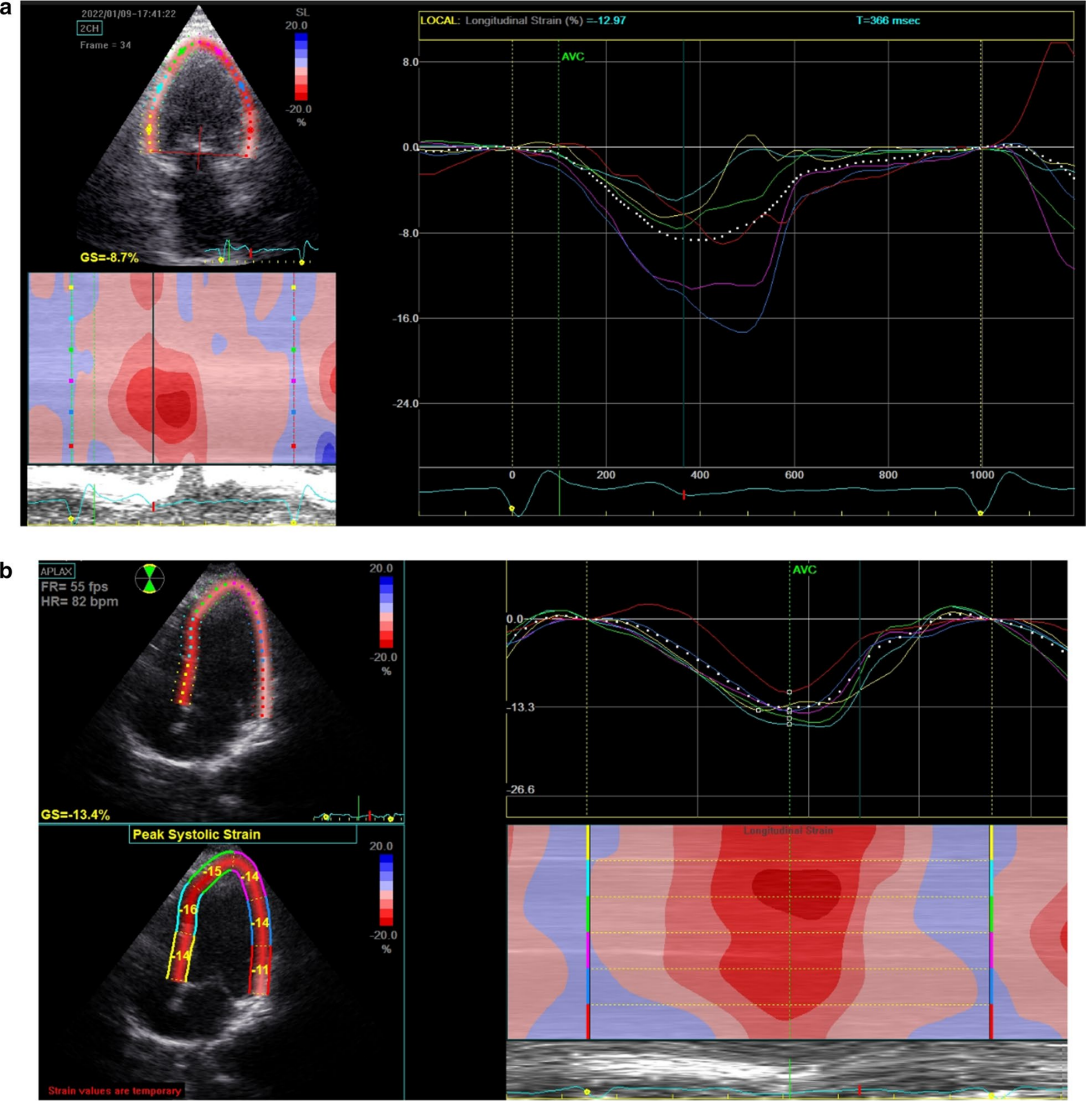
**a** Average GLS for the patient in Fig. 5 before CRT implantation. **b** Average GLS for the same patient in Fig. 5 after CRT implantation that showed significant improvement

*ECG and echocardiogram* A standard supine 12-lead ECGs (10 mm/mV amplitude, 25 mm/s speed) recorded before and after CRT implantation. Echocardiographic study was acquired using GE machine version Vivid E95 and S3 adult probe at baseline and at 6 months after device implantation to evaluate LV end-diastolic volume (LVEDV), LV end-systolic volume (LVESV) (Fig. 3) and LVEF by biplane Simpson’s method and also speckle tracking echocardiography for assessment of GLS (Figs. 4 and 5) and GCS [19] (according to The European society of cardiology current and evolving Echocardiographic techniques, 2011), The same probe used to obtain the usual 2D images was used for recording the 2D loops used for STE. Image acquisition: Images were acquired during breath holds with stable electrocardiographic recordings and ECG gated, digitally stored for offline analysis. At least 3 sinus rhythm cycles were stored in order to perform offline STE analysis. The LV was imaged in the apical four, three and two chamber views for assessment of GLS and again LV was imaged in apex, mid and base in parasternal short axis view for assessment of GCS. An optimal frame rate of 60 to 80 frames per second was obtained by adjusting the sector width and depth of the image to focus on the LV.

*Methods of analysis* Analysis was done offline using the Echo-PAC software. Left ventricular strain was analyzed using conventional two-dimensional echocardiographic grayscale apical views and short axis images. The region of interest was obtained by tracing the LV endocardial borders in a still frame. The automated software program was used to calculate the frame-to-frame displacements of speckle pattern within the region of interest throughout the cardiac cycle [19].

Two different electrophysiology specialists analyzed the ECG criteria, and two different operators did the standard echocardiographic study as well as the loops analysis for STE; each one of them was randomly assigned to a number of patients from both groups and was blinded from the patient’s data to avoid bias.

Patients had a follow-up of 6 months after device implantation; patients with a reduction in LVESV by 15% assessed by 2D TTE, improvement of EF by 5% using biplane Simpson’s and improvement of one grade in NYHA class were classified as responders; all three criteria had to be fulfilled for defining CRT responders. We used combined clinical and echocardiographic parameters for the assessment of CRT response to increase specificity not only echocardiographic parameters as used by Mi Young Park in his study in 2012. STE was used as secondary end point for observing the changes in GLS and GCS in responders group as well as the non-responders group, yet STE was not used in the criteria of assessment of CRT response [20].

### Statistical analysis

Sample size calculation was done using G* power software version 3.1.0; the primary objective of the current study was to compare LVESV between the two groups assuming alpha error of 0.5 and 80% power; and a sample size of 26 cases per group was needed for standardized effect size of 0.7 in the primary outcome.

Data were collected, revised, coded, and entered to the Statistical Package for Social Science (IBM SPSS) version 23. The quantitative data were presented as mean, standard deviations and ranges when parametric and median, inter-quartile range (IQR) when data found nonparametric. Also, qualitative variables were presented as number and percentages.

The comparison between groups with qualitative data was done by using Chi-square test.

The comparison between two groups with quantitative data and parametric distribution was done by Independent *t*-test, while with non-parametric data was done by using Mann–Whitney test.

The confidence interval was set to 95% and the margin of error accepted was set to 5%. So, the *P*-value was considered significant as the following:

*P* > 0.05: Non-significant, *P* < 0.05: Significant, *P* < 0.01: Highly significant.

## Results

### Baseline characteristics

The study included 67 patients, 39 males (58.2%) and 28 females (41.8%); their age ranged between 52 and 74 years, with mean age of (62.57 ± 5.84) years; their BMI ranged between (24–46) with mean (30.93 ± 4.54); 40 patients had diabetes (59.7%), while 44 patients had hypertension (65.7%); 57 patients had eGFR more than 60 ml/hr (85.1%), while 10 patients had e GFR 30–60 ml/hr (14.9%); 34 patients had dilated-non-ischemic cardiomyopathy (50.7%), while 33 patients had ICM 49.3%.

Fifty-one patients were receiving ACEI (76.1%), while only seven patients were receiving ARNI (10.4%), all patients were receiving BBs while only 64 patients were receiving diuretics (95.5%), and 44 patients were receiving MRAs (65.7%), while 8 patients were receiving SGLT2 inhibitors (11.9%), as shown in Table 1.

**Table 1:** Baseline characteristics of the study population, ECG criteria and echocardiographic criteria before CRT implantation

	No. = 67
*Age*	
Mean ± SD	62.57 ± 5.84
Range	52–74
*Gender*	
Male	39 (58.2%)
Female	28 (41.8%)
*BMI*	
Mean ± SD	30.93 ± 4.54
Range	24–46
*Diabetes*	
Yes	40 (59.7%)
*HTN*	
Yes	44 (65.7%)
*CKD*	
eGFR > 60	57 (85.1%)
eGFR 30–60	10 (14.9%)
*Cardiomyopathy*	
DCM	34 (50.7%)
ICM	33 (49.3%)
*ACEI*	
Yes	51 (76.1%)
*ARNI*	
Yes	7 (10.4%)
*BB*	
Yes	67 (100.0%)
*Diuretics*	
Yes	64 (95.5%)
*MRAs*	
Yes	44 (65.7%)
*SGLT2 inh*	
Yes	8 (11.9%)
*QRS duration (msec)*	
Mean ± SD	148.66 ± 22.42
Range	130–200
*Non-Strauss/Strauss*	
Non-Strauss	37 (55.2%)
Strauss	30 (44.8%)
Range	5–15
*Pre TR*	
Mild	51 (76.1%)
Moderate	12 (17.9%)
Severe	4 (6.0%)
*Pre FAC*	
Normal	57 (85.1%)
Impaired	10 (14.9%)
*Pre RVSP (mmhg)*	
Mean ± SD	33.49 ± 9.25
Range	30–60
*Responders*	
Non-responders	16 (24.2%)
Responders	50 (75.8%)
*GLS improvement*	
Median (IQR)	− 4 (− 6 to − 3)
Range	− 11–0
*GCS improvement*	
Median (IQR)	− 5 (− 7 to − 3)
Range	− 10–0
*Post TR*	
Mild	50 (75.8%)
Moderate	12 (18.2%)
Severe	4 (6.1%)
*Post FAC*	
Normal	57 (86.4%)
Impaired	9 (13.6%)
*Post RVSP*	
Mean ± SD	34.38 ± 8.21
Range	30–65
*6-month hospitalization*	
Yes	25 (37.3%)
*Mortality*	
Yes	1 (1.5%)

Angiotensin-converting enzyme inhibitor, angiotensin receptor neutrilypsin inhibitor, beta-blockers, body mass index, chronic kidney disease, dilated cardiomyopathy, hypertension, ischemic cardiomyopathy, mineralocorticoids receptor antagonists, standard deviation, fractional area change, right ventricle systolic pressure, standard deviation, tricuspid regurgitation, fractional area change, global longitudinal strain, global circumferential strain, right ventricle systolic pressure, standard deviation, tricuspid regurgitation

### Pre-operative ECG and pre-operative echocardiography:

The mean QRS duration was 148.66 ± 22.42 ms and that range was 130–200 ms. In 37 patients ECG showed non-Strauss criteria of LBBB (55.2%), while 30 patients ECG showed Strauss ECG criteria of LBBB (44.8) %).

Before CRT implantation 51 patients included in the study had mild TR (76.1%), while 12 patients had moderate TR (17.9%) and only 4 patients had severe TR (6%).

Before CRT implantation 57 patients had normal RV systolic functions assessed by Fractional area change with 85.1%, while 10 patients had impaired systolic functions with 14.9%. Before CRT implantation the RVSP in the patients ranged between 30 and 60 mmhg mean ± SD (33.49 ± 9.25), as shown in Table 1.

### Six-month follow-up data:

Fifty patients matched the criteria of responders to CRT after 6 months (75.8%), while only 16 patients did not match the criteria (24.2%).

Global longitudinal strain improvement ranged between 0 and − 11 with median − 4, while GCS improvement ranged between 0 and − 10 with median − 5 in the whole study population.

After CRT implantation 50 patients included in the study had mild TR (75.8%), while 12 patients had moderate TR (18.2%) and only 4 patients had severe TR (6.1%).

Fifty-seven patients had normal RV systolic functions after CRT implantation, assessed by FAC (86.4%), while 9 patients had impaired systolic functions (13.6%).

Also, after CRT implantation the RVSP in the patients ranged between 30 and 65 mmhg with mean SD (34.38 ± 8.21).

Twenty-five patients were hospitalized for Decompensated heart failure in the 6-month follow-up post-CRT implantation with a percentage of (37.3%).

Only one patient died 4 months post-CRT implantation in the hospital due to acute heart failure, as shown in Table 1.

### The descriptive data comparison between Strauss and non-Strauss group:

There was no significant difference regarding age, gender, BMI between Strauss and non-Strauss group.

Also, there was no significant difference regarding DM and cardiomyopathy between the two groups, but hypertension was more common among Strauss group (*P* value = 0.001). CKD patients were higher in non-Strauss group (*P* value = 0.009).

Both groups showed no statistical significance as regards receiving antifailure measures as ACEI, ARNI, BBs, MRAs, and SGLT2 inhibitor. Only the use of diuretics was higher in the non-Strauss group (*P* value = 0.049).

The mean QRS duration was higher in Strauss group (166.33 ± 22.05 ms vs 134.32 ± 7.65 ms in non-Strauss group with *P* value = < 0.001), and this may be due to the more strict criteria of selection among the Strauss group.

Comparing the NYHA class, there was a statistically significant improvement among the Strauss group (*P* value = 0.023).

Regarding the echocardiographic parameters, the percentage of ESV reduction in echocardiographic assessment after 6 months was significantly higher in Strauss group (*P* value = 0.023), the percentage of EF improvement in echocardiographic assessment using biplane Simpson's method after 6 months was highly significant in Strauss group (*P* value = < 0.001), and also the improvement of Global longitudinal strain and global circumferential strain assessed by 2D speckle tracking echocardiography was highly significant among Strauss group (*P* value = < 0.001).

The 6-month hospitalization and mortality showed no statistical significance between the two groups, as shown in Table 2.

**Table 2:** The descriptive data, clinical and echocardiographic parameters comparison between Strauss and non- Strauss group

	Non-Strauss	Strauss	Test value	*P*-value	Sig.
	No. = 37	No. = 30			
*Age*					
Mean ± SD	62.97 ± 5.69	62.07 ± 6.08	0.629•	0.532	NS
Range	52–74	52–73			
*Gender*					
Male	24 (64.9%)	15 (50.0%)	1.505*	0.220	NS
Female	13 (35.1%)	15 (50.0%)			
*BMI*					
Mean ± SD	30.00 ± 3.25	32.07 ± 5.60	− 1.890•	0.063	NS
Range	24–37	24–46			
*Diabetes*					
Yes	22 (59.5%)	18 (60.0%)	0.002*	0.964	NS
*HTN*					
Yes	18 (48.6%)	26 (86.7%)	10.622*	0.001	HS
*CKD*					
eGFR > 60	33 (89.2%)	24 (80%)	9.395*	0.009	HS
eGFR30-60	4 (10.8%)	6 (20.0%)			
*Cardiomyopathy*					
DCM	21 (56.8%)	13 (43.3%)	1.194*	0.274	NS
ICM	16 (43.2%)	17 (56.7%)			
*ACEI*					
Yes	27 (73.0%)	24 (80.0%)	0.450*	0.502	NS
*ARNI*					
Yes	4 (10.8%)	3 (10.0%)	0.012*	0.914	NS
*BB*					
Yes	37 (100.0%)	30 (100.0%)	NA	NA	NS
*Diuretics*					
Yes	37 (100.0%)	27 (90.0%)	3.873*	0.049	S
*MRAs*					
Yes	23 (62.2%)	21 (70.0%)	0.451*	0.502	NS
*SGLT2 inh*					
Yes	4 (10.8%)	4 (13.3%)	0.100*	0.752	NS
*QRS duration (msec)*					
Mean ± SD	134.32 ± 7.65	166.33 ± 22.05	− 8.251•	< 0.001	HS
Range	130–160	140–200			
*NYHA improvement*					
No	10 (27.8%)	6 (20.0%)	9.562*	0.023	S
One-class	23 (63.9%)	12 (40.0%)			
Two-class	3 (8.3%)	11 (36.7%)			
Three-class	0 (0.0%)	1 (3.3%)			
*ESV reduction*					
Mean ± SD	15.63 ± 2.34	17.92 ± 4.47	3.352•	0.023	S
Range	5–18	5–27			
*EF improvement by Simpson*					
Mean ± SD	5.81 ± 0.85	9.42 ± 3.05	5.801•	< 0.001	HS
Range	5–8	6–15			
*GLS improvement*					
Median (IQR)	− 3 (− 4 to − 3)	− 6 (− 7 to − 4)	4.732^≠^	< 0.001	HS
Range	− 6–0	− 11 to − 2			
*GCS improvement*					
Median (IQR)	− 3 (− 4 to − 2)	− 7 (− 8 to − 5)	4.919^≠^	< 0.001	HS
Range	− 7–0	− 10 to − 2			
*6-month Hosp*					
Yes	13 (35.1%)	12 (40%)	0.676*	0.879	NS
*Mortality*					
Yes	1 (2.7%)	0(0%)	0.823*	0.364	NS

Angiotensin converting enzyme inhibitor, angiotensin receptor neutrilypsin inhibitor, beta-blockers, body mass index, chronic kidney disease, dilated cardiomyopathy, hypertension, ischemic cardiomyopathy, mineralocorticoids receptor antagonists, standard deviation, end systolic volume, ejection fraction, global longitudinal strain, global circumferential strain, New York Heart Association, right ventricle systolic pressure, standard deviation, tricuspid regurgitation. *: Chi-square test; •: Independent t-test; ≠: Mann-Whitney test

### Comparison of baseline characteristics and preoperative ECG between responder and non-responders group:

There was no statistical difference regarding age, gender, and BMI between the responders and non-responders group, there was no statistical significance regarding HTN and diabetes among the two groups, and CKD patients eGFR above 60 ml/hour were higher among the Responders group, while patients having eGFR below 60 ml/hour were higher among non-responders group. (*P* value = < 0.001).

The percentage of DCM patient was significantly higher among the responders group, while the percentage of ICM patients was higher among the non-responders group (*P* value = 0.003).

The percentage of patients receiving ACEI and MRAs were statically higher among the responders group, while there was no statistical significance in patients receiving BBs, diuretics, and SGLT2 inhibitors between the two groups.

The mean QRS duration was longer among Responders group (mean = 152.80 ± 24.25 ms), while the mean QRS duration among the non-responders group was 135.00 ± 5.16 ms (*P* value = 0.005).

The overall response rate to CRT implantation did not statistically differ among Strauss and non-Strauss group, while there was statistically significant improvement with each parameter among the Strauss group as mentioned before, as shown in Table 3.

**Table 3:** The descriptive data, clinical parameters comparison between responders and non-responders group

	Non-Responders	Responders	Test value	*P*- value	Sig.
	No. = 16	No. = 50			
*Age*					
Mean ± SD	62.75 ± 5.05	62.52 ± 6.17	0.135•	0.893	NS
Range	53–69	52–74			
*Gender*					
Male	12 (75.0%)	26 (52.0%)	2.625*	0.105	NS
Female	4 (25.0%)	24 (48.0%)			
*BMI*					
Mean ± SD	31.94 ± 4.82	30.54 ± 4.46	1.069•	0.289	NS
Range	26–43	24–46			
*Diabetes*					
Yes	10 (62.5%)	29 (58.0%)	0.102*	0.750	NS
*HTN*					
Yes	9 (56.2%)	34 (68.0%)	0.737*	0.391	NS
*CKD*					
eGFR > 60	7 (43.8%)	50 (100%)	32.602*	< 0.001	HS
eGFR30-60	9 (56.2%)	0 (0.0%)			
*Cardiomyopathy*					
DCM	3 (18.8%)	31 (62.0%)	9.078*	0.003	HS
ICM	13 (81.2%)	19 (38.0%)			
*ACEI*					
Yes	7 (43.8%)	43 (86.0%)	11.781*	0.001	HS
*ARNI*					
Yes	0 (0.0%)	7 (14.0%)	2.506*	0.113	NS
*BB*					
Yes	16 (100.0%)	50 (100.0%)	–	–	–
*Diuretics*					
Yes	16 (100.0%)	47 (94.0%)	1.006*	0.316	NS
*MRAs*					
Yes	4 (25.0%)	40 (80.0%)	16.500*	< 0.001	HS
*SGLT2 inhibitor*					
Yes	2 (12.5%)	6 (12.0%)	0.003*	0.957	NS
*QRS duration (msec)*					
Mean ± SD	135.00 ± 5.16	152.80 ± 24.25	− 2.901•	0.005	HS
Range	130–140	130–200			
*Non-Strauss/Strauss*					
Non-Strauss	10 (62.5%)	26 (52.0%)	0.539*	0.463	NS
Strauss	6 (37.5%)	24 (48.0%)			
Range	–	5–15			

Body mass index, chronic kidney disease, dilated cardiomyopathy, hypertension, ischemic cardiomyopathy, standard deviation, angiotensin converting enzyme inhibitor, angiotensin receptor neutrilypsin inhibitor, beta-blockers, body mass index, chronic kidney disease, dilated cardiomyopathy, mineralocorticoids receptor antagonists, standard deviation, number. *: Chi-square test; •: Independent t-test

### Comparison between responders and non-responders group regarding echocardiographic parameters and 6-month hospitalization:

Regarding echocardiographic parameters before CRT implantation. Three patients had severe TR and 9 patients had moderate TR among the non-responders group, while only 3 patients had moderate TR and 47 patients had mild TR in the responders group with *P* value = < 0.001).

Eight patients had normal right ventricle systolic functions pre-CRT implantation assessed by fractional area change and eight patients had impaired RV systolic functions in the non-responders group, while 49 patients had normal RV systolic functions and only one patient had impaired systolic functions among the responders group (*P* value =  < 0.001).

Mean RVSP pre-CRT implantation was 43.44 ± 10.28 among the non-responders group, while the mean RVSP was 30.92 ± 5.00 among the responders group (*P* Value = < 0.001).

There was no statistically significant difference in grade of TR, right ventricle systolic functions and RVSP after 6 months of CRT implantation.

There was a statistically significant difference regarding hospitalization with Decompensated heart failure during 6 months post-CRT implantation (*P* value = < 0.001), as shown in Table 4.

**Table 4:** Comparison between responders and non-responders group regarding echocardiographic parameters and 6-month hospitalization

	Non-responders	Responders	Test value	*P*- value	Sig.
	No. = 16	No. = 50			
*Pre TR*					
Mild	4 (25.0%)	47 (94.0%)	33.677*	< 0.001	HS
Moderate	9 (56.2%)	3 (6.0%)			
Severe	3 (18.8%)	0 (0.0%)			
*Pre FAC*					
Normal	8 (50.0%)	49 (98.0%)	23.714*	< 0.001	HS
Impaired	8 (50.0%)	1 (2.0%)			
*Pre RVSP (mmhg)*					
Mean ± SD	43.44 ± 10.28	30.92 ± 5.00	6.577•	< 0.001	HS
Range	30–60	1–40			
*Post TR*					
Mild	4 (25.0%)	46 (92.0%)	29.627*	< 0.001	HS
Moderate	9 (56.2%)	3 (6.0%)			
Severe	3 (18.8%)	1 (2.0%)			
*Post FAC*					
Normal	8 (50.0%)	49 (98.0%)	23.714*	< 0.001	HS
Impaired	8 (50.0%)	1 (2.0%)			
*Post RVSP*					
Mean ± SD	45.25 ± 10.50	30.90 ± 2.19	9.195•	< 0.001	HS
Range	30–65	30–40			
*6-month Hosp*					
Yes	15 (93.8%)	9 (18.0%)	41.083*	< 0.001	HS

fractional area change, right ventricle systolic pressure, standard deviation, Tricuspid regurgitation. *: Chi-square test; •: Independent t-test

## Discussion

In 2019, Zeyu Jiang et al. showed in his study that was done on 181 patients with LBBB morphology where he divided the patient into three groups, a group with non-Strauss LBBB, a group with complete LBBB (Strauss criteria) and a third group for patients with *Q* wave in lead *I* and aVL; then the complete LBBB group was further divided to three groups according to presence or absence of S wave in V5–V6; response after a six-month follow-up was defined in his study as improvement in ESV by 15% only; and the results showed that the group with S wave in lead v6 showed higher incidence for hospitalization and higher mortality risk [21].

Also his study showed that non-SLBBB patients with narrower QRS duration had less favorable CRT response and these results were concordant with our study.

Zeyu Jiang data concurred with the previous results that patients with q-SLBBB had a significantly lower rate of CRT response than patients with CLBBB, but his data also showed that patients with Strauss ECG criteria have a statistically better response to CRT and that was discordant with our data that showed no statistical significance in overall improvement. This may be explained by the difference in the parameters of assessment of CRT response. Also, the aforementioned study was a retrospective study, while our study was a prospective study with follow-up after 6 months [21].

Bertaglia et al. [22] demonstrated that the Strauss criteria did not actually predict better CRT response.

Our study showed that patients with longer QRS duration had better CRT response and these results were concordant with Pieter van der Bijl in 2017, his study was a registry about HF patients who underwent CRT implantation and the patients were divided into three groups: patients with QRS duration less than 150 ms, patients with LBBB and QRS duration more than 150 ms and patients with non-LBBB and QRS duration more than 150 ms [23].

Also, the echocardiographic criteria of response to CRT in that study were similar to ours regarding reduction in ESV and improvement in EF.

Petter Storsten study demonstrated that patients with LBBB demonstrated distinct early systolic shortening in the RV free wall, so in patients with LBBB, RV systolic function was maintained by vigorous contraction in the late-activated LV lateral wall, which pushed the septum toward the RV so LBBB reduces workload on the RV free wall because of delayed activation of the LV lateral wall and abnormal septal motion [24].

Restoring septal and LV function by CRT increases the workload in RV free wall, and this explains why patients with RV failure respond poorly to CRT.

In our study patients with moderate to severe TR, impaired RV functions and high pulmonary artery pressure showed poor response to CRT in 6-month follow-up in concordance with Petter Storsten et al. study [24].

Also, our study demonstrated that CRT does not improve RV functions as the patient had almost the same efficiency of RV systolic functions before and after CRT implantation.

In our study we found patients with DCM are better responders than ICM patients and that may be related to the pathology of the cardiomyopathy and the ischemic scar effect on the synchronization of both ventricles and this was concordant with the results of Takaya Y et al. [13].

Patients receiving ACEI showed better response after 6-month follow-up after CRT implantation.

Also all patients on ARNI were responders, but the number of patients did not reach the statistical power to show the significance of ARNI treatment.

In our study, all patient underwent STE for assessment of GLS and GCS before and after CRT implantation and the results revealed that all responders showed significant improvement of Strain values.

In 2018, Zibire et al. showed in his study that STE can be used as a strong and valid parameter for CRT response [25].

In our study, there was no difference of statistical significance in baseline characteristics (age, gender, BMI) among the Strauss and non-Strauss group; also both groups showed no difference neither regarding cardiomyopathy type nor receiving Antifailure treatment. The mean QRS duration was higher in Strauss group due to the stricter Criteria of LBBB.

There was no significant difference in overall CRT response and 6-month hospitalization between Strauss and non-Strauss groups. However, the individual components of CRT response inclusive of improvement in NYHA class, LV EF, ESV, and strain improved significantly in Strauss group versus non-Strauss.

The most important outcome of our study is that the benefits of CRT implantation are not limited to patients with Strauss LBBB, and that patients with non-Strauss ECG criteria of LBBB could have a better quality of life and better improvement in symptoms with CRT implantation.

## Conclusions

The Strict Criteria of LBBB (Strauss criteria) do not affect the overall response to CRT implantation, the 6-month hospitalization or mortality, yet they have a positive effect on the degree of improvement of each of the clinical and echocardiographic parameters. Also, patients with poor RV functions have poor response to CRT.

### Limitations and recommendations

The study was done on a limited number of cases (70 cases). A larger sample size will demonstrate more accurate data. We recommend using more complex and accurate tools for assessment of cardiac volumes and myocardial dysynchrony as cardiac MRI. Our study was done in a single center, so multi-centered studies are recommended in the future.

## Data Availability

The datasets used and analyzed during the current study are available from the corresponding author on reasonable request.

## Abbreviations

ACEI: Angiotensin-converting enzyme inhibitor
ARNI: Angiotensin receptor neutrilypsin inhibitor
BMI: Body mass index
CKD: Chronic kidney disease
CRT: Cardiac resynchronization therapy
DM: Diabetes mellitus
EF: Ejection fraction
EDV: End-diastolic volume
ESV: End-systolic volume
FAC: Fractional area change
GCS: Global circumferential strain
GLS: Global longitudinal strain
HTN: Hypertension
ICM: Ischemic cardiomyopathy
LBBB: Left bundle branch block
LV: Left ventricle
MRA: Mineralocorticoids receptor antagonists
M-mode: Motion mode
NYHA: New York Heart Association
RVSP: Right ventricle systolic pressure
STE: Speckle tracking echocardiography
TR: Tricuspid regurgitation
TTE: Trans-thoracic echocardiography
2D: Two-dimensional
3D: Three-dimensional

## References

[1] Linde C, Mealing S, Hawkins N, Eaton J, Brown B, Daubert JC (2011). Cost-effectiveness of cardiac resynchronization therapy in patients with asymptomatic to mild heart failure: Insights from the European cohort of the REVERSE (resynchronization reverses remodeling in systolic left ventricular dysfunction). Eur Heart J.

[2] Ojo A, Tariq S, Harikrishnan P, Iwai S, Jacobson JT (2017). Cardiac resynchronization therapy for heart failure. Interv Cardiol Clin.

[3] Moss AJ, Hall WJ, Cannom DS (2009). Cardiac-resynchronization therapy for the prevention of heart-failure events. N Engl J Med.

[4] Kronborg MB, Nielsen JC, Mortensen PT (2010). Electrocardiographic patterns and long-term clinical outcome in cardiac resynchronization therapy. Europace.

[5] Leeters IPM, Davis A, Zusterzeel R (2016). Left ventricular regional contraction abnormalities by echocardiographic speckle tracking in combined right bundle branch with left anterior fascicular block compared to left bundle branch block. J Electrocardiol.

[6] Strauss DG, Selvester RH, Wagner GS (2011). Defining left bundle branch block in the era of cardiac resynchronization therapy. Am J Cardiol.

[7] Auricchio A, Lumens J, Prinzen FW (2014). Response to Kenneth C. Bilchick, MD, MS. Circ Arrhythmia Electrophysiol.

[8] Sweeney MO, Van Bommel RJ, Schalij MJ, Borleffs CJW, Hellkamp AS, Bax JJ (2010). Analysis of ventricular activation using surface electrocardiography to predict left ventricular reverse volumetric remodeling during cardiac resynchronization therapy. Circulation.

[9] Poposka L, Boskov V, Risteski D (2018). Electrocardiographic parameters as predictors of response to cardiac resynchronization therapy. Open Access Maced J Med Sci.

[10] Seo Y, Ito H, Nakatani S (2011). The role of echocardiography in predicting responders to cardiac resynchronization therapy - results from the Japan cardiac resynchronization therapy registry trial (J-CRT). Circ J.

[11] Rickard J, Bassiouny M, Cronin EM (2011). Predictors of response to cardiac resynchronization therapy in patients with a non-left bundle branch block morphology. Am J Cardiol.

[12] Steffel J, Robertson M, Singh JP (2015). The effect of QRS duration on cardiac resynchronization therapy in patients with a narrow QRS complex: a subgroup analysis of the EchoCRT trial. Eur Heart J.

[13] Takaya Y, Noda T, Nakajima I (2014). Electrocardiographic predictors of response to cardiac resynchronization therapy in patients with intraventricular conduction delay. Circ J.

[14] Heart failure/transplant ventricular asynchrony predicts a better outcome (2005) N Engl J Med 352:1539–49.

[15] Salukhe TV, Francis DP, Sutton R (2003). Comparison of medical therapy, pacing and defibrillation in heart failure (COMPANION) trial terminated early; combined biventricular pacemaker-defibrillators reduce all-cause mortality and hospitalization. Int J Cardiol.

[16] Zusterzeel R, Curtis JP, Caños DA (2014). Sex-specific mortality risk by QRS morphology and duration in patients receiving CRT: results from the NCDR. J Am Coll Cardiol.

[17] Perrin MJ, Green MS, Redpath CJ (2012). Greater response to cardiac resynchronization therapy in patients with true complete left bundle branch block: a PREDICT substudy. Europace.

[18] Ponikowski P, Voors AA, Anker SD (2016). ESC guidelines for the diagnosis and treatment of acute and chronic heart failure: the task force for the diagnosis and treatment of acute and chronic heart failure of the European society of cardiology (ESC). Developed with the special contribution. Eur J Heart Fail.

[19] Maréchaux S, Guiot A, Castel AL (2014). Relationship between two-dimensional speckle-tracking septal strain and response to cardiac resynchronization therapy in patients with left ventricular dysfunction and left bundle branch block: a prospective pilot study. J Am Soc Echocardiogr.

[20] Park MY, Altman RK, Orencole M (2012). Characteristics of responders to cardiac resynchronization therapy: the impact of echocardiographic left ventricular volume. Clin Cardiol.

[21] Jiang Z, Qiu Y, Qian Z (2020). An S wave in ECG lead V6 predicts poor response to cardiac resynchronization therapy and long-term outcome. Hear Rhythm.

[22] Bertaglia E, Migliore F, Baritussio A (2017). Stricter criteria for left bundle branch block diagnosis do not improve response to CRT. PACE - Pacing Clin Electrophysiol.

[23] van der Bijl P, Khidir M, Leung M (2017). Impact of QRS complex duration and morphology on left ventricular reverse remodelling and left ventricular function improvement after cardiac resynchronization therapy. Eur J Heart Fail.

[24] Storsten P, Aalen JM, Boe E (2020). Mechanical effects on right ventricular function from left bundle branch block and cardiac resynchronization therapy. JACC Cardiovasc Imaging.

[25] Fulati Z, Liu Y, Sun N (2018). Speckle tracking echocardiography analyses of myocardial contraction efficiency predict response for cardiac resynchronization therapy. Cardiovasc Ultrasound.

